# Association between Breastmilk LC PUFA, Carotenoids and Psychomotor Development of Exclusively Breastfed Infants

**DOI:** 10.3390/ijerph16071144

**Published:** 2019-03-30

**Authors:** Monika A. Zielinska, Jadwiga Hamulka, Iwona Grabowicz-Chądrzyńska, Joanna Bryś, Aleksandra Wesolowska

**Affiliations:** 1 Department of Human Nutrition, Faculty of Human Nutrition and Consumer Sciences, Warsaw University of Life Sciences—SGGW, 159 Nowoursynowska St., 02-776 Warsaw, Poland; monika_zielinska@sggw.pl; 2 Psychological-Pedagogical Counselling Centre No 12, Dzielna St. 1a, 00-162 Warsaw, Poland; iwona.chadrzynska@onet.pl; 3 Department of Chemistry, Faculty of Food Sciences, Warsaw University of Life Sciences—SGGW, Nowoursynowska St. 166, 02-787 Warsaw, Poland; joanna_brys@sggw.pl; 4 Laboratory of Human Milk and Lactation Research at Regional Human Milk Bank in Holy Family Hospital, Department of Neonatology, Faculty of Health Sciences, Medical University of Warsaw, 63A Zwirki i Wigury St., 02-091 Warsaw, Poland; aleksandra.wesolowska@wum.edu.pl

**Keywords:** arachidonic acid (AA), α-linolenic acid (ALA), docosahexaenoic acid (DHA), linoleic acid (LA), lutein, β-carotene, lycopene, human milk, mature milk, motor development

## Abstract

The first months of infant life are crucial for proper neurodevelopment, which may be influenced by several factors, including nutrition and nutrients (e.g., long-chain polyunsaturated fatty acids (LC PUFA) and carotenoids) of which the concentration in breastmilk is diet-dependent. This study analysed the relationship between the average concentrations of selected LC PUFA and carotenoids in breastmilk samples from the first and third months of lactation and the psychomotor development of exclusively breastfed infants at the sixth month of life. Infant psychomotor development was assessed using the Children Development Scale (DSR). The average age of infants during the assessment was 6.6 ± 0.2 months and 30.9 ± 3.8 years for mothers (*n* = 39 mother–infant pairs). The average concentration of docosahexaenoic acid (DHA) was 0.50% of fatty acids. The average concentration of carotenoids was 33.3 nmol/L for β-carotene, 121 nmol/L for lycopene and 33.3 nmol/L for lutein + zeaxanthin. The total results of the Performance scale and Motor subscale were 39 centiles and 4.1 points, respectively. Adjusted multivariate regression models revealed associations between breastmilk DHA and motor development (*β* = 0.275; *p* ≤ 0.05), α-linolenic acid (ALA; *β* = 0.432; *p* ≤ 0.05), *n*-3 LC PUFA (*β* = 0.423; *p* ≤ 0.05) and β-carotene (*β* = 0.359; *p* ≤ 0.05). In addition, an association between the Perception subscale and DHA was observed (*β* = 0.316; *p* ≤ 0.05; model 2). There were no significant associations between the overall Performance scale scores. Due to the positive association between concentrations of *n*-3 LC PUFA (ALA and DHA) and β-carotene in breastmilk and infant motor development, it is important to provide these nutrients with breastmilk. According to the diet-dependent concentration of these compounds in breastmilk, breastfeeding mothers should have a diet abundant in dietary sources of these nutrients, e.g., fish, nuts, seeds, vegetable oils, vegetables and fruits.

## 1. Introduction

Breastfeeding influences infant development and future health. For infants, breastmilk should be the only food in the first six months of life, which supplies not only all essential nutrients but also bioactive factors [1,2]. Breastfeeding is linked to positive health outcomes in mothers and infants and to positive cognitive outcomes in infants [3]. Moreover, the associations between breastfeeding and neurodevelopment are dose-dependent (both for exclusivity and the duration of breastfeeding) and stronger in preterm infants. This link is observed in both developing and developed countries [4,5,6,7,8,9]. Previous studies have shown that, during the first three postnatal months, the most rapid metabolic changes occur in the brain, as well as the rapid synthesis of brain membranes, synaptogenesis, intragyral connectivity and lamination. The brain volume during this time increases from approximately 28–43% of the adult brain [10,11,12]. As far as is known, breastfed infants not only had higher cognitive abilities but also had better brain structural development (e.g., greater grey and white matter volume [13,14]) and physiological activity [15,16,17] compared to formula-fed infants. It has been suggested that this may be related to breastmilk nutrients, especially long-chain polyunsaturated fatty acids (LC PUFA) and carotenoids (especially lutein). However, the results are still inconclusive [18,19,20,21,22]. 

LC PUFA, especially docosahexaenoic acid (DHA), are structural constituents of cell membrane phospholipids (phosphatidylcholine, phosphatidylethanolamine, phosphatidylserine and phosphatidylinositol), especially in the central nervous system [23]. DHA constitutes over 90% of all *n*-3 PUFA in the brain and 10–20% of its total lipids [24]. A previous study using a baboon model showed that prenatal and early postnatal development are a period of rapid DHA accumulation in brain tissue, especially in grey matter areas associated with attention, motor control and sensory integration [25]. Newborns and infants are dependent on maternal DHA status due to a very low DHA conversion from α-linolenic acid (<1%). During pregnancy, DHA is provided via placental transport and later in breastmilk or infant formula if an infant is not breastfed [26,27]. Human studies have shown that infants fed with formula without DHA have a 15% lower concentration of DHA in the brain cortex [28,29]. Intervention studies conducted among formula-fed infants have shown that the DHA fortification of infant formulae improves developmental outcomes in infants, including cognition and visual acuity [30]. Arachidonic acid (AA) is another LC PUFA considered essential for infant development [31]. AA accumulates in the brain during early development and is responsible for neuronal firing, long-term potentiation and hippocampal plasticity. AA is also provided to infants via breastmilk or infant formula, and its breastmilk concentration is usually higher and more stable compared with DHA [31]. Previous studies demonstrated that not only DHA is important for proper psychomotor development but also the balance between AA and DHA is crucial [32]. A systematic review showed that an AA-to-DHA ratio greater than 1:1 was associated with better cognitive outcomes [33].

In recent years, there has also been increased interest in the role of carotenoids, especially lutein (which accumulates in the brain tissue) in brain development and functions [34]. In vitro and in vivo studies have also shown that lutein can improve cell communication and protects neurons against oxidative damage [34]. Studies conducted among adults, the elderly and preadolescent children revealed that carotenoid status is positively associated with cognitive abilities [34,35]. A study of the brain tissue of infants who died in the first 1.5 years of life revealed that lutein was the predominant carotenoid, accounting for 59% of total brain carotenoids [36]. Researchers also showed that formula-fed preterm decedents had a lower brain lutein concentration compared with breastfed infants; however, this difference was not observed in term infants. Other studies found that the lutein concentration in infant brains was correlated with several metabolic pathways in the brain, including lipid, energy, brain osmolytes and amino acid-derived neurotransmitters [37]. A study conducted among nonhuman primate infants fed a formula with low or high carotenoid concentrations, showed that a carotenoid-supplemented formula led to increased lutein in brain tissues, especially in the occipital cortex, hippocampus and the striatum [38]. It was also shown that formula supplementations (with carotenoids) increased brain carotenoids. Moreover, lutein absorption was higher from breastmilk than infant formula [39,40]. However, there is a lack of studies investigating the association between carotenoids and the psychomotor outcomes in infants.

The aim of this study was, therefore, to investigate the relationship between the average concentrations of selected LC PUFA and carotenoids in breastmilk samples from the first and third months of lactation and the psychomotor development of exclusively breastfed infants at the sixth month of life.

## 2. Materials and Methods

### 2.1. Ethical Approval

This study obtained approval from the Ethics Committee of the Medical University of Warsaw in 2015, Resolution No. AKBE/139/15. A written consent agreement was obtained from all participants, and the study was conducted in compliance with the Helsinki Declaration.

### 2.2. Study Design and Sample Characteristics

**Figure 1:**
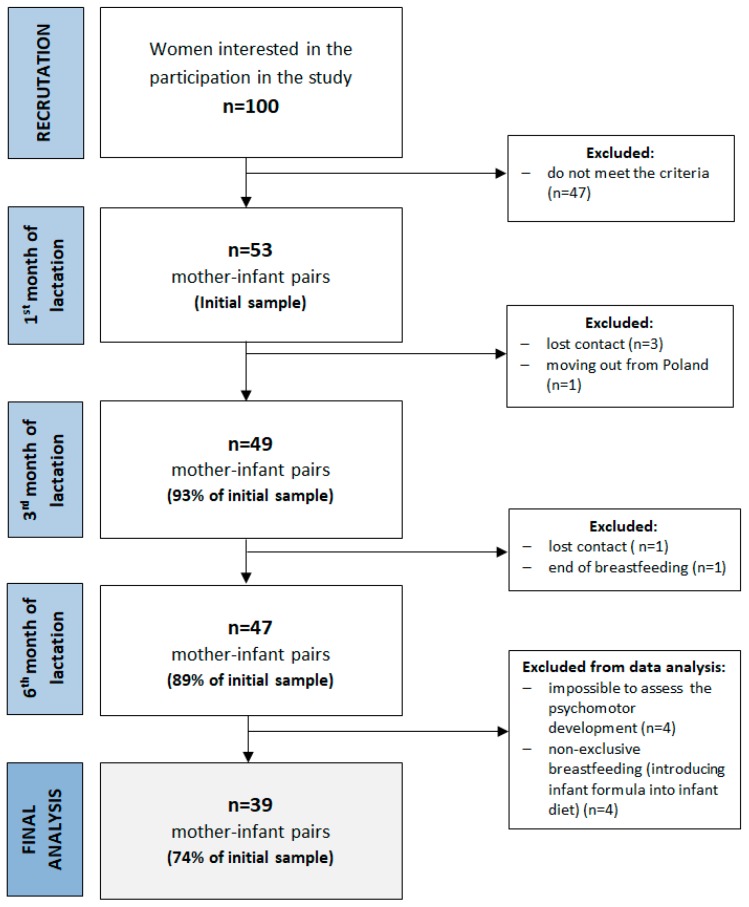
A flowchart of the study design and study sample collection.

The present study was conducted in the central urban area of Poland between 2015–2017. The study consisted of three study visits (first, third and sixth months of lactation). More details about the study design, sample collection and methods used have been described previously [41]. Among the inclusion criteria were maternal age ≥ 19 y, infant age ≤ 6 weeks, singleton birth and plan to exclusively breastfeed for six months. The exclusion criteria included maternal chronic diseases, pregnancy complications, alcohol or tobacco use, vegan diet, preterm birth, low birth weight, birth defects and low milk supply. Fifty-three healthy, exclusively breastfed infants and their mothers participated in the first study visit, and forty-seven completed the study (Figure 1). Exclusive breastfeeding was defined according to the WHO (no other food or drink, not even water, except breastmilk for 6 months of life, but the infant was allowed to receive vitamins, minerals and medicines) [42]. During participation in the study, four infants were supplemented with formula, and were excluded from the final analysis. No other infants received water or other food. In addition, the assessment of psychomotor development at the third study visit (at the sixth month of lactation) was impossible for four infants due to sleeping or crying. The final analysis was conducted among thirty-nine infants. Detailed characteristics of the study group are shown in Table 1 and Table 2. The average age of mother participants was 30.9 ± 3.8 years and most of them were multiparous. The infants were born in the 39.3 ± 1.2 weeks of pregnancy and most of them were girls (54%). The average results of the Edinburgh Postpartum Depression Scale (EPDS) were 4.99 ± 3.04, and the Perceived Stress Scale (PSS-10) was 21.2 ± 3.3 points. 

### 2.3. Breastmilk Collection

Breastmilk samples were collected at the first-, third- and sixth-month visits at home after given instruction. Mothers collected the same amount of pre- and post-feeding milk at four time periods (06:00–12:00; 12:00–18:00; 18:00–24:00; and 24:00–06:00) and transported the samples kept cool at 4 °C. At the laboratory, the same amount of each breastmilk sample was mixed and vortexed. The pooled sample was stored in 2 mL and 10 mL polypropylene tubes at −80 °C for later analysis of carotenoids and fatty acid profile, respectively. During all procedures, the exposure of the samples to temperature, light and air was minimized [41].

### 2.4. Breastmilk Carotenoid Analysis

Carotenoid analyses were conducted using the high-performance liquid chromatography (HPLC) method as detailed previously [41]. Before analysis, the breastmilk samples were saponified based on the modified method by Macias and Schweigert [43]. The concentrations of selected carotenoids (β-carotene, lycopene and lutein + zeaxanthin) were determined using a Shimadzu HPLC system (Japan: 2 LC-20AD pumps, CMB-20A controller system, SIL-20AC autosampler, UV/IS SPD-20AV detector, CTD-20AC controller) using C18 Synergi Fusion-RP 80i columns (250 × 4.60 mm, Phenomenex, CA, USA). The concentration of carotenoids was assessed based on standard curves prepared with Sigma Aldrich standards (catalogue numbers: β-carotene C4582, lutein X6250, lycopene L9879, Merck KGaA, Darmstadt, Germany) and expressed in nmol/L.

### 2.5. Breastmilk Fatty Acid Profile Analysis

The fatty acid profiles in breastmilk samples were determined by a gas chromatography (GC) analysis of fatty acid methyl esters. Prior to the analysis, fat from the breastmilk was extracted using the Folch procedure, which had been slightly modified by Boselli et al. [44,45]. The obtained fats were then analysed for fatty acid composition. The fats first underwent saponification and esterification with potassium hydroxide in methanol to obtain fatty acid methyl esters (FAME). The fatty acids contained in the fats were determined with the GC technique as described previously [46]. All of the used solvents and reagents were purchased from Avantor Performance Materials Poland S.A. (Gliwice, Poland), whereas the FAME chemical standard was purchased from Sigma Aldrich (Sigma Aldrich, Merck KGaA, Darmstadt, Germany).

### 2.6. Assessment of Infant Psychomotor Development

**Figure 2:**
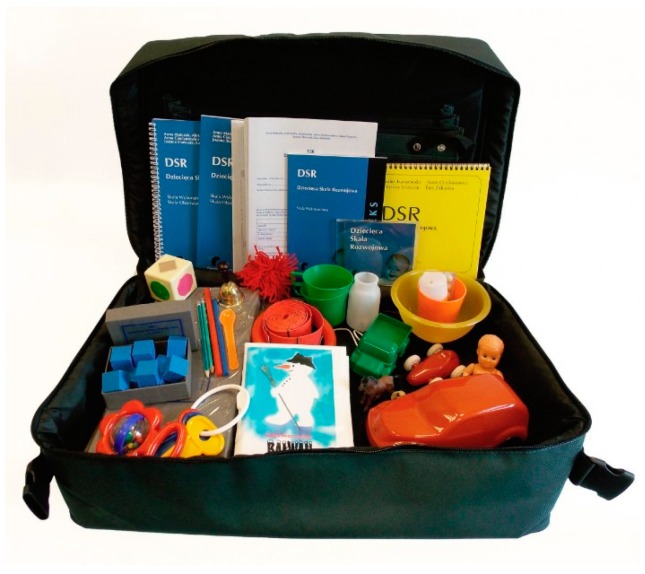
The Children Development Scale DSR Kit [50].

The infant psychomotor development was assessed by a qualified child psychologist. For the assessment of psychomotor development, the Children Development Scale (DSR) was used [47,48,49]. This scale is well-standardized and normalized in the Polish population and is used for psychomotor development assessment in children aged 2–36 months (Figure 2). It provides information on the level and profile of child psychomotor development and consists of two scales: a Performance Scale with 10 subscales (Manipulation, Perception, Scribble and Drawing, Building Blocks, Similarities, Memory, Speech and language, Social behaviour and Motor skills) and an Observational Scale, which assesses four temperament traits (Vigor, Adaptability, Regularity and Sensitivity). In this study, we used only subtests of a Performance Scale suitable for infants age (Manipulation, Perception, Memory, Speech and language, Social behaviour and Motor skills), and the total results are expressed as centiles.

### 2.7. Covariates 

During the analyses, the following potential confounders were collected or measured in three areas: parental (maternal and paternal age and education level, marital status, maternal psychological status and maternal nutrition), household (household income), pregnancy-related (gestational age, birth parameters and gender) and infant-related (children anthropometric parameters and nutrition). Maternal nutrition was assessed by a qualified dietician using the 12-month Food Frequency Questionnaire (FFQ) at the first month of lactation and a 3-day dietary record at the third and sixth months of lactation. Maternal psychological status was assessed by a psychologist at each study visit using the Polish version of the short, validated questionnaires EPDS [51] and PSS-10 scales [52,53]. The data on maternal prepregnancy and pregnancy body mass and infant birth parameters were collected retrospectively. Maternal and infant current body mass and height or length were measured at each study visit. Detailed information on study design and data collection have been described previously [41].

### 2.8. Statistical Analysis

For variables assessed at all three study visits (breastmilk linoleic acid (LA), α-linolenic acid (ALA), docosahexaenoic acid (DHA), eicosapentaenoic acid (EPA), arachidonic acid (AA), β-carotene, lycopene and lutein + zeaxanthin and maternal psychological status), the mean value from the first and second study visits was calculated and used for further analysis. The continuous variables were presented as mean values and standard deviation (SD) or confidence intervals (CI) with minimum and maximum values. The normality of variable distribution was checked with the Shapiro–Wilk test. A linear regression analysis was used to investigate the associations between breastmilk nutrients and psychomotor development at the sixth month of life. Both univariate and multivariate models were calculated separately for each nutrient (adjusted for infant age, maternal age and education, child gender, maternal psychological status, birthweight and number of children in the household). The results were reported as regression coefficients, along with their 95% confidence intervals (CI) and statistical significance level (*p*-value). For the models, the R^2^ parameters and statistical significance levels were calculated and reported. All analyses were performed using the Statistica 13.1 software (Dell Inc., TA, USA). For all tests, *p* ≤ 0.05 was considered significant.

## 3. Results

### 3.1. Selected LC PUFA and Carotenoids in Breastmilk

The average concentrations of DHA, AA and ALA were 0.50%, 0.19% and 1.2% of fatty acids in the first and third months of lactation, respectively (Table 3). The average *n*-6 to *n*-3 and AA-to-DHA ratio were 6.5 and 0.44, respectively. The highest concentration of determined carotenoids in breastmilk samples obtained at the first and third month of lactation was observed for lycopene, whereas β-carotene and lutein + zeaxanthin had much lower concentrations. 

### 3.2. Results of Infant Psychomotor Development Assessment

Table 4 provides the results of the DSR Performance Scale assessing the infant psychomotor development at the sixth month of life. The lowest mean score was observed in subscale memory (0.8 points, 95% CI 0.6–0.9), and the highest was for the subscale assessing social development (6.7 points, 95% CI 6.4–7.1). The mean total score of the DSR Performance scale was 39 centiles (95% CI 35–43) and ranged from 16 to 74 centiles. There were no differences in any DSR Performance subscale or total scores of the Performance scale between boys and girls or mode of delivery (vaginal or caesarean section).

On the manipulation subscale, *n* = 10 (26%) infants were in a low category and *n* = 29 (74%) were in an average category. On the social behaviour subscale, most infants were in an average or high subscale (*n* = 32; 82%; *n* = 3; 8%) and only four (10%) infants were in the low category. The highest results were obtained in the motor skills subscale, where all infants were in the average (*n* = 24; 62%) or high (*n* = 15; 38%) categories. Most infants on the overall Performance Scale were in the average (*n* = 26; 67%) or above average (*n* = 2; 5%) categories, whereas 28% (*n* = 2; 5% and *n* = 9; 23%, respectively) were in the low or moderately low categories.

### 3.3. Associations between Selected LC PUFA and Carotenoids in Breastmilk and Infant Psychomotor Development

Table 5 presents the results of the multivariate linear regression analysis of the associations between the breastmilk concentration of selected LC PUFA and carotenoids and the results of infant motor development. The results of other Performance Scale Subscales and the total result are presented in Table S1. Model 1 unadjusted for any infant or maternal factors revealed significant positive associations between the motor skills score and breastmilk DHA (*β* = 0.358; *p* ≤ 0.05) and β-carotene (*β* = 0.348; *p* ≤ 0.05). After an adjustment for infant age, gender, maternal age, education and maternal psychological status (model 2), these results remained significant, but significant associations were also found with ALA (*β* = 0.518; *p* ≤ 0.01), *n*-3 LC PUFA (*β* = 0.501; *p* ≤ 0.01) and *n*-6 to *n*-3 ratio (*β* = −0.320; *p* ≤ 0.05). After another adjustment for infant birthweight and number of children in the household, the associations for DHA (*β* = 0.275; *p* ≤ 0.05), ALA (*β* = 0.432; *p* ≤ 0.05), *n*-3 LC PUFA (*β* = 0.423; *p* ≤ 0.05) and β-carotene (*β* = 0.359; *p* ≤ 0.05) were significant. For other subscales, we observed significant associations in the univariate analysis for lycopene and the Manipulation subscale (*β* = 0.348; *p* ≤ 0.05) and for DHA and the Perception subscale in model 1 and 2 (*β* = 0.381; *p* ≤ 0.05; *β* = 0.316; *p* ≤ 0.05, respectively; Table S1).

## 4. Discussion

In this study, a positive association was found between the concentration of several LC PUFA (DHA, ALA and *n*-3 PUFA) and β-carotene in breastmilk and motor development but not in overall psychomotor development at the sixth month of life of healthy, term-delivered, exclusively breastfed infants. No associations were observed between concentration of lutein + zeaxanthin and lycopene in breastmilk and motor development. 

In the study, it was found that a concentration of *n*-3 LC PUFA, ALA and DHA in breastmilk during the first three months of life is associated with better motor development at the sixth month of life even after an adjustment for confounders. Previous observational studies reported no associations between breastmilk DHA and ALA concentrations and subsequent psychomotor development (Table 6) [54,55,56,57]. Studies from Spain and France showed that the total *n*-3 PUFA concentrations in colostrum may improve cognitive development, whereas *n*-6 LC PUFA may decrease it [21,55,57]. This association may be explained by the competition of *n*-3 and *n*-6 LC-PUFA for the same enzymes, which may result in a decrease in the biosynthesis of *n*-3 LC PUFA [58]. In addition, a higher concentration of circulating linoleic acid could decrease the uptake of DHA into the brain [59]. Gustafsson et al. [60] found positive associations between the DHA concentration in colostrum and infant cognitive development. In addition, Cheatham and Sheppard [20] found that exclusively breastfed infants had better recognition memory at the sixth month of life when milk contained more DHA and choline (not only DHA). These discrepancies in study results may be related to the limited sensitivity of global tests used to assess the psychomotor development of healthy, term infants [54]. Moreover, the LC PUFA levels in breastmilk observed in the current study were different from the worldwide average; DHA and ALA levels were higher, AA and LA were lower and the EPA levels were similar [61,62]. The other possible explanation is the difference in the breastfeeding assessments between studies, especially since the duration and exclusivity of breastfeeding is not taken into account. Without this data, a precise estimation of infant exposure to the breastmilk bioactive components is impossible due to the fact that exclusively and mixed-fed infants have a different intake of breastmilk [55]. 

The current study showed no association between breastmilk lutein + zeaxanthin and psychomotor development at the sixth month of life. However, Cheatham and Sheppard [20] found positive associations between lutein and choline concentrations and infant recognition memory assessed at the sixth month of life and breastmilk but no associations for lutein itself. According to our knowledge, the current study and a study by Cheatham and Sheppard [20] are the only studies which have been conducted among infants. Moreover, studies conducted among adults have shown that lutein + zeaxanthin supplementation improves neural processing speed and efficiency in healthy young adults and that brain levels of lutein and β-carotene were correlated with cognitive performance in the elderly [34]. A study conducted among preadolescent children showed that macular pigment optical density, which is a noninvasive indicator of lutein status, was correlated with academic performance in children [63]. However, a study conducted among healthy younger children (5.6–5.9 years) did not find any associations between serum lutein and cognitive performance [64]. The lack of any associations obtained in our study may be a result of the limited sensitivity of tests used to assess psychomotor development. In addition, the population in our study, as well as in a study by Mulder et al. [64], were particularly well-nourished and had a low risk of nutritional deficiency. This may decrease the ability to detect associations between lutein intake and nutrition status and cognitive performance because lutein may have the largest functional effects in the population with relative nutritional deficiency [65]. 

The current study showed an association between breastmilk β-carotene and motor development at the sixth month of life. β-carotene is one of the major brain carotenoids, but its concentration in the infant's brain is much lower than lutein [36]. As far as is known, there are no observational studies investigating the associations between β-carotene intake or nutritional status and cognitive performance or psychomotor development in children. The concentration of β-carotene observed in the current study was lower and less varied compared to lycopene and lutein + zeaxanthin. Despite that, in our previous paper we found that its breastmilk concentration is diet-dependent [41]. However, Gossage et al. [66] did not observe any increase in the β-carotene concentration in breastmilk after its supplementation. They noted that this may be related with saturation of breastmilk with β-carotene. Moreover, previous studies have been shown that β-carotene uptake and cleavage was reduced when dietary vitamin A was optimal and its metabolism depended on a variety factors (e.g., genetic, health and dietary) [67]. Our study group was well-nourished, and we observed that a narrow range of the concentration of β-carotene in breastmilk may indicate the existence of a metabolism mechanism influencing β-carotene transport to the breastmilk. However, despite the small diversity in the concentration of β-carotene in breastmilk, we observed an association with infant motor development significant after adjustment for infant age, gender, maternal age, education level, psychological status (EPDS and PSS-10 scores), birthweight and the number of children in the household. Further research is needed to explain the associations between β-carotene concentration in breastmilk and infant motor development.

It is worth noting that both *n*-3 LC PUFA and carotenoid concentration in breastmilk are diet-dependent [41,68,69,70]. The DHA concentration in breastmilk may be increased through the consumption of fish [70] or dietary supplementation, both during lactation and pregnancy [71,72]. The previous studies showed that a DHA dose of 1.0–7.5 g/day was safe for mothers and infants and was linked with other health benefits in adults, including cardiovascular and brain function in adults [73,74]. Carotenoid concentration in breastmilk could also be influenced through dietary intervention [75,76,77,78] and was related to its dietary intake and vegetables and fruit consumption [41,79]. During pregnancy and the lactation period, a diet abundant in vegetables and fruit should be recommended not only because of the increase in breastmilk carotenoids but also because of the other health benefits associated with their consumption [80]. A diet positively influencing the levels of *n*-3 LC PUFA and carotenoids in breastmilk might be the Mediterranean diet, which is rich in vegetables and fruits, nuts and seeds, whole grains and fish and seafoods, foodstuffs typically containing a high amount of these nutrients and bioactives. However, the detailed impact of this diet on breastmilk composition is still under investigation [81].

The exact mechanisms of breastfeeding in promoting psychomotor and cognitive development have been widely discussed. It has been emphasized that not only breastmilk nutrients and bioactive factors but also other maternal factors related to breastfeeding, including socioeconomic or psychosocial factors, play a crucial role in supporting psychomotor and cognitive development [82,83]. Infant neurocognitive development may also be influenced by both inherent factors (e.g., genetic, prenatal, hormonal and health-related factors) and environmental factors, including environmental pollution [84,85]. Previous studies have shown that breastfeeding mothers are more likely to have a higher IQ, educational level and income and are more likely to provide a cognitively stimulating environment for their infants and that these factors may be responsible for the observed effects, not breastfeeding itself [83,86]. A systematic review conducted by Walfish et al. [82] found that studies of the associations between breastfeeding and IQ provided an almost equal number of positive and negative associations and that many of them were not effectively adjusted for potential confounders. However, meta-analyses conducted by Horta et al. [9], which also included studies adjusted for the home environment, showed that breastfeeding was associated with 3.44 points more in IQ tests in childhood and adolescence, and these associations remained significant after the adjustment for maternal IQ (2.62, 95% CI 1.25–3.98). Lucas et al. [87] and Isaacs et al. [13] conducted studies among preterm infants who were fed breastmilk during hospitalization. Both studies showed that preterm infants fed with breastmilk obtained better results in IQ tests [13,87]. Our results, as well as the studies mentioned above, support the hypothesis that the nutritional properties of breastmilk have a biological effect on psychomotor and cognitive development. Several studies have shown that the results of early developmental assessment and IQ may predict cognitive abilities in further life [88,89,90]. From a public health perspective, considering the other health outcomes for infants and mothers provided by breastfeeding, its promotion may be a very cost-effective strategy to significantly improve a child’s health and development.

### Strengths and Limitations

The strength of this study is that it used the DSR test for psychomotor development assessment, which is characterized by good psychometric properties and is normalized for the Polish population [47]. In addition, we collected breastmilk samples at two times (the first and third month of lactation) and assessed infant psychomotor development at the sixth month of life. Moreover, during breastmilk collection, we used a well-defined and clear protocol of breastmilk sample collection to minimize daily and intra-feeding differences in breastmilk component concentrations described in literature [91,92]. An analysis was conducted on the associations between breastmilk nutrients and psychomotor development only among exclusively breastfed infants to minimize the effect of different exposures to breastmilk nutrients between exclusively breastfed and mixed-fed infants. 

However, this study has several limitations. First, the study group is small, as well as well-nourished, which may decrease the chance of finding positive associations between assessed nutrients and development outcomes. Moreover, the study sample was characterized by a very good socioeconomic status and an education level that was higher than the national average. In Poland, 54% of 24–34-year-old women have tertiary education compared to 34% of men in the same age group. These rates are higher than the Organisation for Economic Co-operation and Development (OECD) average from 2017 (51%) [93]. Moreover, in recent years in Poland, there has been a steady increase in women who gave birth and who have a university education (from 6% in 1990 to 52% in 2017) especially in cities (59%) [94]. The higher education level observed in our study may be associated with the fact that the study was conducted in Warsaw, the largest city in Poland, with a higher education and economic level than other areas in Poland. Moreover, women who exclusively breastfed their infants and were interested in participation in this study might have a higher education level. The study group also had a high rate of caesarean section (38%), slightly higher than the national average since 2010 [95], although it was lower than the 44% observed in southern Poland in 2013–2014 [96], which is consistent with data obtained by the Childbirth with Dignity Foundation in 2015 for all of Poland (43%) [97]. Secondly, a psychological test was used to assess psychomotor development, whereas the use of other methods (e.g., electrophysiological methods) may be more effective to assess brain and cognitive development. However, these methods are more expensive than psychomotor tests. Moreover, an assessment under laboratory conditions using special equipment is necessary, although it may induce stress in infants. In addition, since an analysis of psychomotor development was conducted only at the sixth month of life and the positive effect of nutrients may be revealed more distinctly in later development stages, a longer follow-up period would be desired. In addition, we did not assess maternal IQ as one of the potential confounders.

## 5. Conclusions

In this study, we assessed the association between selected LC PUFA and carotenoids in breastmilk in the first and third months of life and the psychomotor development of exclusively breastfed infants at the sixth month of life. Although we found a positive association between the concentration of *n*-3 LC PUFA, ALA, DHA and β-carotene breastmilk and motor development, no associations were found for overall psychomotor development. Due to the positive association between concentrations of *n*-3 LC PUFA (ALA and DHA) and β-carotene in breastmilk and infant motor development, it is important to provide these nutrients with breastmilk. According to the diet-related concentrations of these nutrients in breastmilk, breastfeeding mothers should have a diet abundant in dietary sources of these nutrients, e.g., fish, nuts, seeds, vegetable oils, vegetables and fruits. Moreover, nutritional intervention may be a strategy to improve infant motor development and, in consequence, the health of the next generation. 

## Figures and Tables

**Table 1:** The study group characteristics: continuation variables.

Variable	Mean	SD ^1^	Minimum	Maximum
Maternal age (years)	30.9	3.8	23.0	40.0
Infant age at psychomotor assessment (months)	6.6	0.2	6.0	7.0
Gestational age (weeks)	39.3	1.2	37.0	42.0
Birthweight (g)	3380	361	2870	4240
Weight at 6^th^ month (g)	7725	771	6060	9695
EPDS ^2^ result (points)	4.99	3.04	0.0	11.0
PSS-10 ^3^ result (points)	21.2	3.3	14.0	28.0

**^1^** SD, standard deviation; **^2^** EPDS, Edinburgh Postpartum Depression Scale; **^3^** PSS-10, Perceived Stress Scale.

**Table 2:** The study group characteristics: qualitative variables.

Variable	*n*	%
**Parity**		
- primiparous - multiparous	18 21	46 54
**Maternal education**		
- university	39	100
**Marital status**		
- informal relationship - married	6 33	15 85
**Average income per capita (PLN)**		
- <1000 - 1000–1500 - >1500	4 9 26	11 23 66
**Child’s gender**		
- female - male	21 18	54 46
**Type of delivery**		
- vaginal - caesarean section	24 15	62 38

**Table 3:** The average concentration of selected LC PUFA and carotenoids in breastmilk from the first and third months of lactation.

Breastmilk Composition	Mean	95% CI ^1^	Minimum	Maximum
LA ^2^ (% of fatty acids)	11	9.8–11	7.1	17
ALA^3^ (% of fatty acids)	1.2	1.1–1.4	0.7	2.3
AA ^4^ (% of fatty acids)	0.19	0.18–0.20	0.12	0.31
EPA ^5^ (% of fatty acids)	0.10	0.09–0.11	0.070	0.17
DHA ^6^ (% of fatty acids)	0.50	0.44–0.56	0.21	0.99
LC PUFA ^7^ (% of fatty acids)	14	13–15	9.3	20
*n*-3 LC PUFA (% of fatty acids)	1.8	1.7–2.0	1.0	3.0
*n*-6 LC PUFA (% of fatty acids)	12	11–12	8.0	18
*n*-6/*n*-3 ratio	6.5	5.9–7.1	4.3	15
AA/DHA ratio	0.44	0.39–0.49	0.23	0.85
β-carotene (nmol/L)	33.3	33.0–33.5	32.1	34.6
Lycopene (nmol/L)	121	114–127	84.3	168
L + Z ^8^ (nmol/L)	33.3	25.3–41.3	3.17	123

**^1^** CI, confidence intervals; **^2^** LA, linoleic acid; **^3^** ALA, α-linolenic acid; **^4^** AA, arachidonic acid; **^5^** EPA, eicosapentaenoic acid; **^6^** DHA, docosahexaenoic acid; **^7^** LC PUFA, long-chain polyunsaturated fatty acids; **^8^** L + Z, lutein + zeaxanthin.

**Table 4:** The results of the infant psychomotor assessment at the sixth month of life.

DSR ^1^ Performance Scale Results	Median	Mean	95% CI ^2^	Minimum	Maximum
Manipulation (points)	7.0	6.4	6.0–6.8	3.0	7.0
Perception (points)	5.0	4.7	4.4–5.1	2.0	7.0
Memory (points)	1.0	0.8	0.6–0.9	0.0	1.0
Speech and language (points)	2.0	2.0	1.7–2.3	1.0	5.0
Social behaviour (points)	7.0	6.7	6.4–7.1	4.0	9.0
Motor skills (points)	4.0	4.1	3.8–4. 5	3.0	6.0
**Total result of Performance scale (centiles)**	36	39	35–43	16	74

**^1^** DSR, Children Development Scale; **^2^** CI, confidence intervals.

**Table 5:** The results of the linear regression models of the associations between breastmilk nutrients and the results of the Motor Skills DSR Performance subscale (the other subscales are presented in Supplementary Materials Table S1).

Breastmilk Nutrients	Model 1		Model 2		Model 3	
	β (95% CI ^2^)	R^2^	β (95% CI ^2^)	R^2^	β (95% CI ^2^)	R^2^
LA ^3^	0.042 (−0.290–0.375)	0.00	0.222 (−0.121*–*0.566)	0.30	0.199 (−0.153–0.551)	0.36
ALA ^4^	0.120 (*−*0.211*–*0.451)	0.01	0.518 (0.175*–*0.861) **	0.44 *	0.432 (0.039–0.825) *	0.43 *
AA ^5^	0.107 (*−*0.224*–*0.438)	0.01	0.066 (−0.272*–*0.403)	0.26	0.078 (−0.263–0.419)	0.33
EPA^6^	*−*0.027 (*−*0.360*–*0.306)	0.00	−0.002 (−0.353*–*0.350)	0.26	−0.056 (−0.416–0.305)	0.33
DHA^7^	0.358 (0.046*–*0.669) *	0.13 *	0.359 (0.047*–*0.671) *	0.37 *	0.275 (0.089–0.640) *	0.38 *
LC-PUFA^8^	0.091 (*−*0.241*–*0.422)	0.01	0.295 (−0.045*–*0.635)	0.33	0.255 (−0.099–0.608)	0.38
*n*-3 LC-PUFA	0.238 (*−*0.086*–*0.561)	0.06	0.501 (0.187*–*0.815) **	0.45 **	0.423 (0.048–0.800) *	0.44 *
*n*-6 LC-PUFA	0.052 (*−*0.281*–*0.384)	0.00	0.221 (−0.121*–*0.562)	0.30	0.201 (−0.148–0.550)	0.36
*n*-6/*n*-3 ratio	*−*0.259 (*−*0.581*–*0.062)	0.07	−0.320 (−0.635*–*−0.005) *	0.35 *	−0.015 (−0.422–0.392)	0.37
AA/DHA ratio	−0.265 (−0.591–0.061)	0.07	−0.355 (−0.677*–*−0.032)	0.34	−0.304 (−0.670–0.062)	0.39
β-carotene	0.348 (0.036–0.660) *	0.12 *	0.296 (−0.031*–*0.623) *	0.33 *	0.359 (0.025–0.693) *	0.43 *
Lycopene	0.158 (−0.171–0.487)	0.02	0.050 (−0.304*–*0.403)	0.26	0.023 (−0.380–0.335)	0.33
L + Z ^9^	0.060 (−0.273–0.392)	0.00	0.175 (−0.167*–*0.518)	0.28	0.214 (−0.137–0.566)	0.36

**^1^** DSR, Children Development Scale, results of selected Performance subscales; **^2^** CI, confidence intervals; **^3^** LA, linoleic acid; **^4^** ALA, α-linolenic acid; **^5^** AA, arachidonic acid; **^6^** EPA, eicosapentaenoic acid; **^7^** DHA, docosahexaenoic acid; **^8^** LC PUFA, long-chain polyunsaturated fatty acids; ^9^ L + Z, lutein + zeaxanthin; **Model 1**: unadjusted model; **Model 2**: adjusted for infant age and gender, maternal age, education and psychological status; **Model 3**: model 2 adjusted for birthweight and parity; * *p* ≤ 0.05; ** *p* ≤ 0.01.

**Table 6:** The summarized results of studies on the associations between breastmilk fatty acids, carotenoids and child development.

Study Group (Country; *n*)	Type of Breastmilk	Type of Infant Feeding	Method of Cognitive/Psychomotor Assessment	Age at Assessment	Breastmilk Nutrients	Results	Reference
Canada *n* = 83	Mature milk (1 week; 1 and 3 months)	Exclusive breastfeeding for at least 3 months	Bayley’s II Scales of Childhood Development	6 and 12 months	DHA^1^	- No significant associations	[54]
Sweden *n* = 131	Colostrum (48–96 h) mature milk (3 month)	Any breastfeeding	Wechsler Preschool and Primary Scale of Intelligence-III	6.5 years	LC PUFA^2^	Full scale IQ^3^ and colostrum: - DHA: *β* = 0.916 *p* = 0.005) - AA^4^: *β* = −0.856 *p* = 0.002)	[60]
Spain *n* = 504	Colostrum (48–96 h)	Any breastfeeding	Bayley Scales of Infant Development	14 months	LC PUFA	Total score: - ALA^5^/LA^6^ ratio: *β* = 6.12 (95%CI^7^ 1.63–10.62) - *n*3/*n*6 ratio: *β* = 5.50 (95%CI 1.05–9.94) - EPA^8^/AA ratio: *β* = 4.51 (95%CI 0.125–8.89) - DHA: *β* = 3.44 (95%CI: 0.90–7.78) - total *n*-3 LC PUFA: *β* = 4.85 (95%CI 0.48–9.23)	[55]
Denmark *n* = 53	Mature milk (2 month)	Any breastfeeding	Bayley’s III Scales of Childhood Development	12 months	LC PUFA	Cognitive scale score - AA: *r* = 0.46, *p* = 0.002	[56]
USA *n* = 67	Mature milk (3–5 months)	Exclusive breastfeeding by 6 months	Electrophysiology Methods (event-related potentials, eye movements)	6 months	DHA, lutein, choline	Better recognition memory: - ↑ DHA and ↑choline - ↑ lutein and ↑choline	[20]
France *n* = 652	Colostrum	Ever-exclusive breastfeeding	French Psychomotor Developmental Scale for Early Childhood of Brunet-Lézine; Ages and Stages Questionnaire	2 and 3 years	LC PUFA	Motor development at 2 years: - LA: −0.1 (−0.2–0.0) - total *n*-6 LC PUFA: −0.1 (−0.2–0.0) Cognition at 3 years: - LA: −1.9 (−3.1–0.08) - total *n*-6 LC PUFA: −2.0 (−3.1–0.9)	[57]
France *n* = 1080	Colostrum	Any breastfeeding	Wechsler Preschool and Primary Scale of Intelligence-III	5–6 years	LC PUFA	Verbal IQ: - LA: *β* = −0.06 (−1.1, 0.0) Full scale IQ: - LA ↑ and DHA ↓ vs. ↓ LA and DHA: score ↓ by 3.0 (0.5–5.5)	[21]

^1^ DHA, docosahexaenoic acid; ^2^ LC PUFA, long chain polyunsaturated acids; ^3^ IQ, intelligence quotient; ^4^ AA, arachidonic acid; ^5^ ALA, α-linolenic acid; ^6^ LA, linoleic acid; ^7^ CI, confidence intervals; ^8^ EPA, eicosapentaenoic acid; ↑, higher; ↓, lower.

## Supplementary Materials

The following are available online at https://www.mdpi.com/1660-4601/16/7/1144/s1, Table S1: Results of the linear regression models of the associations between breastmilk nutrients and the results of DSR Performance subscales.

## Abbreviations

AA	arachidonic acid
ALA	α-linolenic acid
CI	confidence intervals
DHA	docosahexaenoic acid
DSR	Children Development Scale
EPA	eicosapentaenoic acid
EPDS	Edinburgh Postpartum Depression Scale
FFQ	Food Frequency Questionnaire
GC	gas chromatography
HPLC	high-performance liquid chromatography
IQ	intelligence quotient
LA	linoleic acid
LC PUFA	Long-chain polyunsaturated fatty acids
L + Z	lutein + zeaxanthin
OECD	Organisation for Economic Co-operation and Development
PSS-10	Perceived Stress Scale
