# Supplementary material for: Association between Breastmilk LC PUFA, Carotenoids and Psychomotor Development of Exclusively Breastfed Infants
Source: Int J Environ Res Public Health. 2019 Mar 30;16(7):1144. doi: 10.3390/ijerph16071144 (PMC6479893; doi:10.3390/ijerph16071144)
Supplement: Supplementary file 1 [file ijerph-16-01144-s001.pdf]

International Journal of
Environmental Research and Public Health

MDPI

Article

Association between breastmilk LC PUFA, carotenoids and psychomotor development of exclusively breastfed infants

Monika A. Zielinska [1], Jadwiga Hamulka [1]*, Iwona Grabowicz-Chądrzyńska [2], Joanna Bryś [3], Aleksandra Wesolowska [4]

Table S1. Results of the linear regression models of the associations between breastmilk nutrients and the results of DSR Performance subscales.

DSR [1] subscale	Breastmilk nutrients	Model 1		Model 2		Model 3	
		β (95% CI [2])	R^2	β (95% CI [2])	R^2	β (95% CI [2])	R^2
Manipulation	LA [3]	-0.180 (-0.507 – 0.148)	0.03	-0.048 (-0.424 - 0.328)	0.16	-0.071 (-0.472 - 0.330)	0.17
	ALA [4]	-0.289 (-0.608 - 0.030)	0.08	-0.181 (-0.595 - 0.232)	0.18	-0.233 (-0.701 - 0.236)	0.19
	AA [5]	-0.190 (-0.517 - 0.137)	0.04	-0.199 (-0.552 - 0.153)	0.19	-0.218 (-0.590 - 0.153)	0.21
	EPA [6]	0.069 (-0.310 - 0.448)	0.01	0.088 (-0.285 - 0.461)	0.16	0.158 (-0.240 - 0.556)	0.18
	DHA [7]	-0.179 (-0.507 - 0.149)	0.03	-0.162 (-0.519 - 0.194)	0.18	-0.231 (-0.645 - 0.183)	0.20
	LC-PUFA [8]	-0.211 (-0.536 - 0.116)	0.04	-0.08 (-0.457 - 0.303)	0.16	-0.105 (-0.512 - 0.303)	0.17
	n-3 LC-PUFA	-0.281 (-0.601 - 0.039)	0.08	-0.189 (-0.571 - 0.194)	0.18	-0.260 (-0.706 - 0.186)	0.21
	n-6 LC-PUFA	-0.176 (-0.503 - 0.152)	0.03	-0.049 (-0.422 - 0.324)	0.16	-0.074 (-0.472 - 0.324)	0.17
	n-6/*n*-3 ratio	0.149 (-0.181 - 0.478)	0.02	0.125 (-0.230 - 0.481)	0.17	0.153 (-0.243 - 0.549)	0.18
	AA/DHA ratio	0.069 (-0.268 - 0.406)	0.00	0.006 (-0.361 - 0.372)	0.15	0.066 (-0.362 - 0.494)	0.17
	β-carotene	-0.185 (-0.512 - 0.143)	0.03	-0.288 (-0.639 - 0.064)	0.23	-0.278 (-0.667 - 0.111)	0.22
	Lycopene	0.348 (0.036 - 0.660) *	0.12 *	-0.303 (-0.662 - 0.056)	0.23	-0.306 (-0.685 - 0.074)	0.24
	L + Z [9]	-0.140 (-0.470 - 0.190)	0.02	-0.144 (-0.512 - 0.223)	0.17	-0.144 (-0.543 - 0.255)	0.18
Perception	LA [3]	-0.239 (-0.563 – 0.084)	0.06	-0.274 (-0.626 - 0.078)	0.26	-0.307 (-0.661 - 0.046)	0.35
	ALA [4]	-0.195 (-0.521 - 0.132)	0.04	-0.224 (-0.623 - 0.175)	0.24	-0.128 (-0.569 - 0.312)	0.29
	AA [5]	-0.162 (-0.491 - 0.167)	0.03	-0.101 (-0.449 - 0.248)	0.21	-0.141 (-0.491 - 0.209)	0.29
	EPA [6]	-0.032 (-0.365 - 0.301)	0.00	0.075 (-0.288 - 0.438)	0.21	0.222 (-0.142 - 0.586)	0.32
	DHA [7]	0.381 (0.073 - 0.689) *	0.15 *	0.316 (0.015 - 0.648) *	0.29 *	-0.235 (-0.618 - 0.148)	0.32
	LC-PUFA [8]	-0.288 (-0.607 - 0.031)	0.08	-0.315 (-0.667 - 0.036)	0.28	-0.332 (-0.690 - 0.026)	0.36
	n-3 LC-PUFA	-0.305 (-0.622 - 0.012)	0.09	-0.292 (-0.654 - 0.071)	0.27	-0.187 (-0.606 - 0.232)	0.30
	n-6 LC-PUFA	-0.248 (-0.571 - 0.075)	0.06	-0.274 (-0.623 - 0.075)	0.26	-0.313 (-0.663 - 0.037)	0.36
	n-6/*n*-3 ratio	0.084 (-0.248 - 0.416)	0.01	0.006 (-0.343 - 0.355)	0.20	-0.135 (-0.503 - 0.234)	0.29
	AA/DHA ratio	0.257 (-0.069 - 0.584)	0.07	0.276 (-0.055 - 0.607)	0.31	0.230 (-0.158 - 0.618)	0.31
	β-carotene	0.027 (-0.306 - 0.360)	0.00	-0.061 (-0.418 - 0.296)	0.20	0.007 (-0.368 - 0.382)	0.28
	Lycopene	-0.003 (-0.336 - 0.330)	0.00	0.013 (-0.353 - 0.380)	0.20	-0.011 (-0.381 - 0.360)	0.28
	L + Z [9]	0.195 (-0.132 - 0.522)	0.04	0.117 (-0.242 - 0.475)	0.21	0.176 (-0.192 - 0.544)	0.30
Memory	LA [3]	0.194 (-0.133 – 0.520)	0.04	0.275 (-0.087 - 0.637)	0.22	0.276 (-0.118 - 0.669)	0.20
	ALA [4]	0.185 (-0.143 - 0.512)	0.03	0.267 (-0.140 - 0.674)	0.20	0.340 (-0.126 - 0.806)	0.20
	AA [5]	0.157 (-0.172 - 0.482)	0.02	0.224 (-0.126 - 0.575)	0.20	0.219 (-0.159 - 0.596)	0.18
	EPA [6]	-0.223 (-0.548 - 0.101)	0.05	-0.164 (-0.533 - 0.205)	0.18	-0.102 (-0.509 - 0.305)	0.15
	DHA [7]	0.063 (-0.270 - 0.395)	0.00	0.179 (-0.18 - 0.535)	0.18	0.244 (-0.176 - 0.663)	0.18
	LC-PUFA [8]	0.203 (-0.123 - 0.529)	0.04	0.3049 (-0.059 - 0.667)	0.23	0.315 (-0.083 - 0.712)	0.21
	n-3 LC-PUFA	0.150 (-0.180 - 0.479)	0.02	0.247 (-0.131 - 0.625)	0.20	0.342 (-0.103 - 0.786)	0.21
	n-6 LC-PUFA	0.197 (-0.130 – 0.523)	0.04	0.279 (-0.080 - 0.638)	0.22	0.278 (-0.112 - 0.668)	0.20
	n-6/*n*-3 ratio	0.026 (-0.307 - 0.359)	0.00	-0.000 (-0.359 - 0.358)	0.16	-0.015 (-0.422 - 0.392)	0.14
	AA/DHA ratio	-0.021 (-0.359 - 0.317)	0.00	-0.049 (-0.419 - 0.322)	0.13	-0.023 (-0.458 - 0.413)	0.14
	β-carotene	0.308 (-0.009 - 0.625)	0.09	0.256 (-0.100 - 0.611)	0.21	0.343 (-0.045 - 0.730)	0.23
	Lycopene	-0.106 (-0.437 - 0.226)	0.01	-0.155 (-0.527 - 0.217)	0.18	-0.162 (-0.562 - 0.238)	0.16
	L + Z [9]	0.097 (-0.234 - 0.429)	0.01	-0.016 (-0.387 - 0.355)	0.16	-0.004 (-0.413 - 0.406)	0.14

**Speech and language**	LA [3]	-0.254 (-0.576 – 0.068)	0.06	-0.313 (-0.675 - 0.049)	0.22	-0.263 (-0.617 - 0.092)	0.23
	ALA [4]	-0.291 (-0.610 - 0.028)	0.09	-0.259 (-0.671 - 0.153)	0.18	-0.163 (-0.597 - 0.271)	0.20
	AA [5]	0.111 (-0.220 - 0.420)	0.01	0.157 (-0.202 - 0.516)	0.16	0.172 (-0.172 - 0.516)	0.18
	EPA [6]	-0.127 (-0.458 - 0.203)	0.02	-0.206 (-0.576 - 0.164)	0.17	-0.239 (-0.597 - 0.119)	0.19
	DHA [7]	-0.121 (-0.452 - 0.210)	0.01	-0.043 (-0.408 - 0.322)	0.17	-0.195 (-0.576 - 0.187)	0.19
	LC-PUFA [8]	-0.254 (-0.576 - 0.068)	0.06	-0.311 (-0.677 - 0.056)	0.22	-0.253 (-0.616 - 0.109)	0.23
	n-3 LC-PUFA	-0.270 (-0.591 - 0.051)	0.07	-0.268 (-0.648 - 0.113)	0.19	-0.221 (-0.632 - 0.190)	0.21
	n-6 LC-PUFA	-0.241 (-0.563 - 0.083)	0.06	-0.300 (-0.657 - 0.065)	0.21	-0.246 (-0.599 - 0.108)	0.23
	n-6/*n*-3 ratio	0.090 (-0.241 - 0.422)	0.01	0.036 (-0.326 - 0.398)	0.14	-0.022 (-0.390 - 0.345)	0.16
	AA/DHA ratio	0.231 (-0.098 - 0.560)	0.05	0.287 (-0.065 - 0.640)	0.22	0.363 (-0.004 - 0.730)	0.25
	β-carotene	0.108 (-0.223 - 0.439)	0.01	0.152 (-0.215 - 0.519)	0.16	0.053 (-0.318 - 0.423)	0.19
	Lycopene	-0.014 (-0.347 - 0.319)	0.00	0.049 (-0.331 - 0.429)	0.14	0.040 (-0.326 - 0.405)	0.15
	L + Z [9]	0.128 (-0.203 - 0.458)	0.02	0.164 (-0.206 - 0.534)	0.16	0.077 (-0.292 - 0.446)	0.18
**Social behaviour**	LA [3]	0.009 (-0.324 – 0.342)	0.00	0.116 (-0.262 - 0.494)	0.15	0.149 (-0.247 - 0.544)	0.19
	ALA [4]	-0.060 (-0.393 - 0.273)	0.00	0.017 (-0.407 - 0.441)	0.14	0.076 (-0.398 - 0.550)	0.17
	AA [5]	0.110 (-0.221 - 0.441)	0.01	0.055 (-0.309 - 0.419)	0.14	0.066 (-0.313 - 0.444)	0.18
	EPA [6]	0.131 (-0.200 - 0.461)	0.02	0.098 (-0.279 - 0.474)	0.15	0.080 (-0.320 - 0.479)	0.18
	DHA [7]	0.008 (-0.325 - 0.341)	0.00	-0.043 (-0.408 - 0.322)	0.14	-0.045 (-0.466 - 0.376)	0.17
	LC-PUFA [8]	0.005 (-0.328 - 0.338)	0.00	0.102 (-0.281 - 0.485)	0.15	0.140 (-0.264 - 0.544)	0.19
	n-3 LC-PUFA	-0.034 (-0.367 - 0.299)	0.00	-0.005 (-0.399 – 0.388)	0.14	0.0345 (-0.420 - 0.489)	0.17
	n-6 LC-PUFA	0.015 (-0.318 - 0.348)	0.00	0.113 (-0.262 - 0.489)	0.15	0.146 (-0.246 - 0.539)	0.19
	n-6/*n*-3 ratio	0.096 (-0.235 - 0.428)	0.01	0.149 (-0.209 - 0.507)	0.16	0.149 (-0.245 - 0.543)	0.19
	AA/DHA ratio	0.205 (-0.126 - 0.536)	0.04	0.177 (-0.187 - 0.541)	0.16	0.228 (-0.189 - 0.645)	0.20
	β-carotene	0.422 (0.120 - 0.724)	0.18	0.478 (0.151 - 0.804)	0.34	0.460 (0.102 - 0.819)	0.34
	Lycopene	0.012 (-0.321 - 0.345)	0.01	0.090 (-0.292 - 0.466)	0.14	0.084 (-0.311 - 0.479)	0.18
	L + Z [9]	0.258 (-0.064 - 0.580)	0.07	0.406 (0.062 - 0.749)	0.27	0.381 (0.009 - 0.752)	0.29
**Total result**	LA [3]	-0.167 (-0.500 – 0.167)	0.03	-0.0912 (-0.486 - 0.303)	0.11	-0.074 (-0.482 - 0.335)	0.14
	ALA [4]	-0.075 (-0.412 - 0.262)	0.01	0.518 (0.175 - 0.861)	0.11	0.184 (-0.297 - 0.665)	0.15
	AA [5]	0.048 (-0.290 - 0.386)	0.00	0.069 (-0.310 - 0.448)	0.11	0.077 (-0.310 - 0.464)	0.14
	EPA [6]	-0.051 (-0.389 - 0.286)	0.00	-0.000 (-0.400 – 0.400)	0.10	-0.014 (-0.424 - 0.396)	0.13
	DHA [7]	-0.179 (-0.511 - 0.153)	0.03	-0.170 (-0.542 - 0.202)	0.13	-0.235 (-0.657 - 0.186)	0.17
	LC-PUFA [8]	-0.169 (-0.502 - 0.164)	0.03	-0.166 (-0.516 - 0.184)	0.11	-0.063 (-0.480 - 0.354)	0.13
	n-3 LC-PUFA	-0.133 (-0.468 - 0.202)	0.02	-0.000 (-0.411 - 0.410)	0.10	0.008 (-0.458 - 0.474)	0.13
	n-6 LC-PUFA	-0.162 (-0.495 - 0.172)	0.03	-0.089 (-0.480 - 0.303)	0.11	-0.070 (-0.476 - 0.335)	0.14
	n-6/*n*-3 ratio	-0.000 (-0.338 - 0.338)	0.00	-0.069 (-0.445 - 0.307)	0.11	-0.077 (-0.485 -0.198)	0.14
	AA/DHA ratio	0.230 (-0.099 - 0.559)	0.05	0.224 (-0.144 - 0.592)	0.15	0.204 (-0.092 - 0.743)	0.19
	β-carotene	0.348 (0.036 - 0.660)	0.18	0.404 (0.047 - 0.7602)	0.24	0.383 (0.000 - 0.765)	0.25
	Lycopene	-0.218 (-0.548 - 0.112)	0.05	-0.164 (-0.555 - 0.227)	0.12	-0.164 (-0.564 - 0.237)	0.15
	L + Z [9]	0.280 (-0.045 - 0.604)	0.08	0.316 (-0.055 - 0.687)	0.19	0.287 (-0.107 - 0.682)	0.20

[1] DSR – Children Development Scale; [2] CI - confidence intervals; [3] LA – linoleic acid; [4] ALA - α-linolenic acid; [5] AA – arachidonic acid; [6] EPA – eicosapentaenoic acid; [7] DHA – docosahexaenoic acid; [8] LC PUFA – long chain polyunsaturated fatty acids; [9] L + Z – lutein + zeaxanthin; **Model 1**: unadjusted model; **Model 2**: adjusted for infant age and gender. maternal age. education and psychological status; **Model 3**: model 2 adjusted for birthweight and parity; * $p \leq 0.05$.

© 2019 by the authors. Submitted for possible open access publication under the terms and conditions of the Creative Commons Attribution (CC BY) license (http://creativecommons.org/licenses/by/4.0/).
